# Portents and Epidemics

**Published:** 1888-09-29

**Authors:** 


### Portents and Epidemics.

In reading classic and mediæval accounts of the plagues and accidents that devastated and depopulated whole continents, our attention is always drawn to the signs and wonders which preceded the calamity. The appearance of new stars, or the disappearance of old ones, cyclones and siroccos, strange appearances in earth and sky—all these things are narrated by the awestruck chronicler as having been sent to warn the people of the wrath of an offended Deity. The inference is that had the nations desisted from their darling sins, their idolatry, their luxury, and their immorality, warned by the strange and supernatural portents, the threatened punishment—the plague, the "sweating sickness," or the small-pox—which was coming on the land would have been averted. Sometimes we incline to think that the chroniclers invented or imagined the warnings after the doom had fallen—it is so easy to recall after the event the little signs unnoticed at the time, which might be taken as predicting it. Still, the old historians may have been right in thinking that, had those they dwelt among repented of their sins, they might have evaded the threatened pestilence. Luxury and self-indulgence are no safeguards against misfortune, but the reverse; they weaken the constitution, paralyse the judgment, destroy the natural courage of man, and so predispose to infection. For body, as well as intellect and soul, "plain living and high thinking" are the best regimen.

But it seems on the whole to have missed the intelligence of our chroniclers that the very presages they felt to be so ominous were in themselves the first signs of the coming plague, not an accidental accompaniment, but an integral part of the infection. The strange winds were plague-laden, the rainstorms ruined the ill-built houses and rotted the vegetation, till what should have been the food of man became his poison. The poor were starved, and on the top of the famine came the plague, not as an additional punishment but as an inevitable consequence.

But sometimes the rain was no common shower, but—so the chroniclers tell us—the colour of blood, to the vulgar imagination nothing less than blood itself. Such a shower fell in England in the year 330; in Silesia, in 786, the water became blood; and we are told that in 800, both in Sicily and England, the wells were turned to blood for the space of fifteen days. This cannot be pure legend, for, as we study the medical history of the middle ages, the accounts of these blood-showers become more minute and elaborate. In 1500 such a shower occurred, but the drops were no longer red only, but white, yellow, grey, and black. The spots rested everywhere—on trees and houses, on dress and food—they did not soak into the substance nor evaporate as rain-drops do, and they appeared in chests where no absolute rain could penetrate. People were overcome with superstitious horror; they thought the whole earth was plague-stricken, and that the Day of Judgment was at hand; therefore they deserted their homes and their duties and fled to the woods, to wander there in aimless fear till death overtook them—a poor preparation for the great tribunal they expected.

But the wiser heads among them did not accept the popular explanation of the weird phenomena. These could not be rain-drops which appeared where no rain fell, though they might be connected with the all-pervading dampness of the air. From these facts a wise physician, George Agricola, deduced the theory that they might be the evidences of a minute, all but microscopic, vegetation. As the idea gained ground the superstitious fear died away, and tentative steps were taken to get rid of and prevent the recurrence of such disastrous phenomena, till, from the first perception of the true connexion between portents and epidemics, arose the still young and growing science of sanitation.

